# Structural properties of magnetic nanoparticles determine their heating behavior - an estimation of the *in vivo* heating potential

**DOI:** 10.1186/1556-276X-9-602

**Published:** 2014-11-05

**Authors:** Robert Ludwig, Marcus Stapf, Silvio Dutz, Robert Müller, Ulf Teichgräber, Ingrid Hilger

**Affiliations:** 1 Department of Experimental Radiology, Division of Diagnostic and Interventional Radiology, University Hospital Jena - Friedrich Schiller University Jena, Forschungszentrum Lobeda, Erlanger Allee 101, D-07747 Jena, Germany; 2 Institute of Biomedical Engineering and Informatics, University of Technology Ilmenau, D-98684 Ilmenau, Germany; 3 Department of Nanobiophotonics, Leibniz Institute of Photonic Technology, D-07745 Jena, Germany

**Keywords:** Immobilization, Specific absorption rate (SAR), Intrinsic loss power (ILP), Magnetic nanoparticles (MNP), Magnetic hyperthermia

## Abstract

Magnetically induced heating of magnetic nanoparticles (MNP) in an alternating magnetic field (AMF) is a promising minimally invasive tool for localized tumor treatment by sensitizing or killing tumor cells with the help of thermal stress. Therefore, the selection of MNP exhibiting a sufficient heating capacity (specific absorption rate, SAR) to achieve satisfactory temperatures *in vivo* is necessary. Up to now, the SAR of MNP is mainly determined using ferrofluidic suspensions and may distinctly differ from the SAR *in vivo* due to immobilization of MNP in tissues and cells. The aim of our investigations was to study the correlation between the SAR and the degree of MNP immobilization in dependence of their physicochemical features.

In this study, the included MNP exhibited varying physicochemical properties and were either made up of single cores or multicores. Whereas the single core MNP exhibited a core size of approximately 15 nm, the multicore MNP consisted of multiple smaller single cores (5 to 15 nm) with 65 to 175 nm diameter in total. Furthermore, different MNP coatings, including dimercaptosuccinic acid (DMSA), polyacrylic acid (PAA), polyethylenglycol (PEG), and starch, wereinvestigated. SAR values were determined after the suspension of MNP in water. MNP immobilization in tissues was simulated with 1% agarose gels and 10% polyvinyl alcohol (PVA) hydrogels.

The highest SAR values were observed in ferrofluidic suspensions, whereas a strong reduction of the SAR after the immobilization of MNP with PVA was found. Generally, PVA embedment led to a higher immobilization of MNP compared to immobilization in agarose gels. The investigated single core MNP exhibited higher SAR values than the multicore MNP of the same core size within the used magnetic field parameters. Multicore MNP manufactured via different synthesis routes (fluidMAG-D, fluidMAG/12-D) showed different SAR although they exhibited comparable core and hydrodynamic sizes. Additionally, no correlation between *ζ*-potential and SAR values after immobilization was observed.

Our data show that immobilization of MNP, independent of their physicochemical properties, can distinctly affect their SAR. Similar processes are supposed to take place *in vivo*, particularly when MNP are immobilized in cells and tissues. This aspect should be adequately considered when determining the SAR of MNP for magnetic hyperthermia.

## Background

Up to now, a huge variation range of magnetic nanoparticle (MNP) formulations have been proposed for magnetic hyperthermia applications. Magnetic hyperthermia is characterized by the production of heat by exposure of the target tissues, previously loaded with MNP, to an alternating magnetic field (AMF) due to magnetization reversal losses [1,2]. To achieve hyperthermic temperatures, there is a necessity of selecting MNP which display high specific absorption rates (SAR) and which are consequently able to generate temperatures above 43°C to efficiently eradicate tumor cells and sensitize them for chemotherapy and/or radiation therapy [3-5].

A common technique to determine the SAR of MNP is the use of calorimetric methods. In this context, MNP are exposed to an AMF and the generation of heat in dependence of the amount of the sample’s iron content is determined. To allow the comparison of SAR values independent of the characteristics of the used AMF, consideration of the intrinsic loss power (ILP) has been suggested [6].

Up to now, the calorimetric assessment of the SAR of MNP has been mainly performed by the use of MNP suspended in water. Basically, ILP values between 2 and 4 nHm^2^/kg for magnetic hyperthermia purposes have been reported [6]. Nevertheless, MNP exhibiting ILP values slightly below these values can still be sufficient for heating in magnetic hyperthermia (ILP values of 1.6 nHm^2^/kg; [7,8]). To our knowledge, the highest ILP values ever reported (23.4 nHm^2^/kg) were observed for magnetosomes with core diameters of 30 nm naturally synthesized by bacteria [9]. Interestingly, the most reported values refer to MNP suspended in water, which in consideration of the *in vivo* situation would, at most, resemble the situation after MNP injection into the bloodstream.

The accumulation of MNP in the tumor is an important precondition for magnetic hyperthermia treatments. In this context, one would expect that a distinct proportion of MNP, either injected intravenously or intratumorally, is immobilized to components of the extracellular matrix and cells (tumor cells, fibroblasts, etc.) of the tumor area [7,10]. Although there are few publications using different substances for immobilizing MNP to simulate the above described *in vivo* situation, this important parameter has poorly been considered up to now. The few reports on the SAR of immobilized MNP used agarose or gelatin to immobilize MNP [7,11]. However, utilization of such substances bears several drawbacks such as insufficient inhibition of Brownian relaxation, most likely due to pore sizes of at least 141 nm (e.g., 1% agarose), and too low melting points (e.g., gelatin) which can also interfere with the degree of MNP immobilization [12,13]. Therefore, the majority of the presently known SAR values of MNP, which have been measured after their suspension in water, represent an overestimation of their ‘real’ *in vivo* SAR.

Further on, no systematic studies on the effects of core size, core clustering, hydrodynamic diameter, surface coating, and *ζ*-potential on the SAR are available, which could help chemists to further optimize their MNP synthesis protocols.

For these reasons, we aimed at investigating the relationships between the SAR and the physicochemical structure of MNP. In this context, we used superparamagnetic iron oxide nanoparticle formulations of different core structures: a) single core MNP (non-clustered, 12 to 15 nm) consisting of only one particle core exhibiting one magnetic domain and b) multicore MNP (clustered MNP, 5 to 175 nm) consisting of multiple cores with single magnetic domains [14]. Although the used multicore MNP can comprise minor ferromagnetic portions based on the width of the MNP’s size distribution, they were categorized as SPIONs. Since it has been shown that core and hydrodynamic sizes of MNP can influence their SAR, we also included MNP with varying core (5 to 175 nm) and hydrodynamic sizes (76 to 210 nm) [6,15]. Additionally, little is known about the impact of the MNP coating, and the resulting *ζ*-potential onto the SAR, therefore, MNP covered with different polymers (dimercaptosuccinic acid (DMSA), polyacrylic acid (PAA), polyethylenglycol (PEG), and starch), and different surface functionalizations (COOH, NH_2_) were included in our investigations.

## Methods

### Magnetic nanoparticles

**Figure 1:**
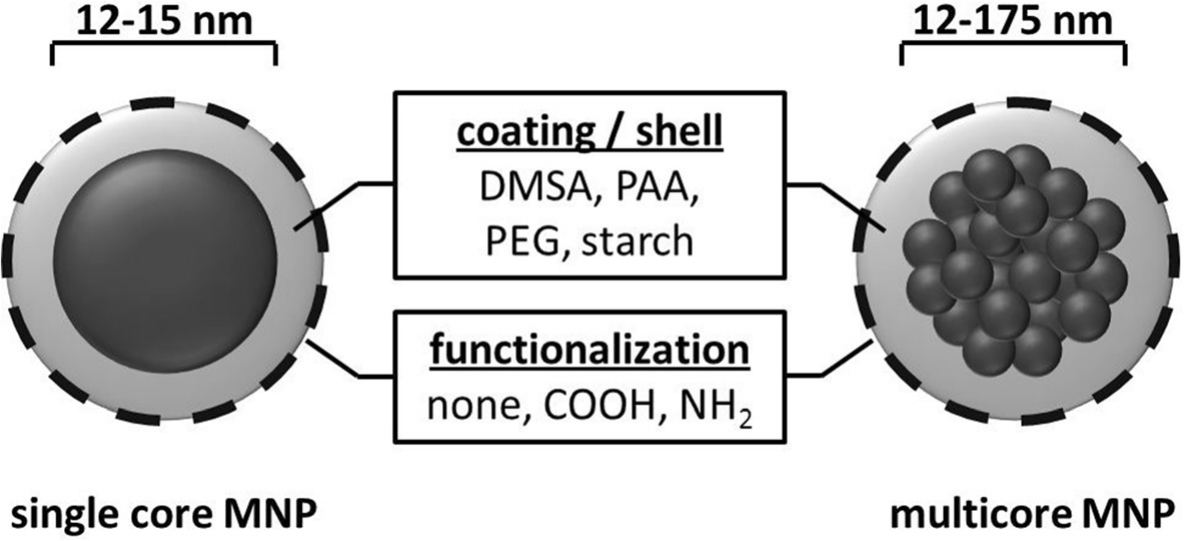
Scheme of idealistic single core and multicore MNP with different core sizes, coatings, and functionalizations.

For our investigations we used magnetite MNP with different features:

1) Single iron oxide core MNP with core diameters of 12 to 15 nm, namely OD15 and MF66 (Figure 1), which were kindly provided from Instituto de Ciencia de Materiales de Madrid (ICMM-CSIC, Campus Universitario de Cantoblanco, Madrid, Spain) and Liquid Research Ltd. (Bangor, United Kingdom) [2]. These MNP exhibited hydrodynamic diameters of 92 to 210 nm and were coated either with DMSA, PAA, or PEG of different molecular weight.2) fluidMAG-D multicore MNP consisted of clustered single iron oxide cores embedded in a dextran matrix. The sizes of the multicore varied between 12 to 175 nm (Figure 1) and the hydrodynamic diameter between 110 and 170 nm. These MNP were coated with starch and kindly provided by chemicell GmbH (Berlin, Germany).3) fluidMAG/12-D multicore MNP consisted of clustered single iron oxide cores embedded in a dextran matrix. The multicore exhibited a size of approximately 70 nm (Figure 1) and the hydrodynamic diameter was measured as 129 nm. The surface coating of these MNP consisted of starch. MNP were kindly provided by chemicell GmbH (Berlin, Germany).

4) nanomag-D MNP consisted of a cluster of single iron oxide cores (5 to 15 nm) embedded in a dextran matrix and leading to overall core sizes of 130 nm. These MNP with hydrodynamic diameters ranging from 156 to 163 nm were coated with a PEG, PEG-NH_2_ (PEG functionalized with amino groups), and PEG-COOH (PEG functionalized with carboxyl groups) shell and obtained from micromod Partikeltechnologie GmbH (Rostock, Germany).

### Determination of MNP size and charge

Determination of MNP core size and shape was performed by high-resolution transmission electron microscopy (HRTEM, microscope Zeiss-CEM 902A (Carl Zeiss AG, Oberkochen, Germany)). Hydrodynamic diameters were obtained via dynamic light scattering (DLS) using a Zetasizer Nano ZS (Malvern Instruments GmbH, Herrenberg, Germany) with a measurement angle of 173° backscatter. DLS data represent the *Z*-average, since it is the most adequate value for size determination provided by this technique. Furthermore, the *ζ*-potential as a measure for the MNP’s surface charge was acquired.

### Specific absorption rate

In accordance with Teran et al. [16], SAR values were determined at initial times directly after applying the AMF (*H* = 15.4 kA/m, *f* = 435 kHz) by the mass-normalized temperature increase. Temperature measurements were performed using a fiber optic temperature sensor and a fiber optic thermometer (TS5 and FOTEMPMK-19, respectively; OPTOCON AG, Dresden, Germany). Iron concentrations of the used samples were determined in triplicates by atomic absorption spectroscopy and the mean values were used to calculate SAR values. For calculation, the following equation

$SAR=c\times \frac{m_{F}}{m_{P}}\times \frac{\Delta T}{\Delta t}\;$, with *c* as the specific sample’s heat capacity, *m*_P_ the MNP’s mass, *m*_F_ the fluid’s mass, and ∆*T*/∆*t* the maximum value of the linear slope at initial times, was used.

Application of MNP *in vivo* leads to their internalization and immobilization in cells and tissues. To simulate this particular *in vivo* situation, MNP were immobilized in either agarose (1% *w*/*v*, agarose NEEO Ultra-Qualität, Carl Roth GmbH, Karlsruhe, Germany) or polyvinyl alcohol (PVA, 10% *w*/*v*, Sigma-Aldrich, St. Lois, USA) in accordance with previous protocols [11,17]. Agarose samples were polymerized for 24 h at 8°C, PVA samples for the same duration at -20°C.

Since SAR values are dependent on the used AC field strength and frequency, system-independent ILP was calculated in accordance with Kallumadil et al. [6] using the following equation: $ILP=\frac{SAR}{H^{2}\times f}$ (*H*: field amplitude, *f*: frequency).

### Magnetic measurements

The quasistatic magnetic properties were investigated using a MicroMagTM3900 (Princeton Measurements Corp., Westerville, OH, USA) vibrating sample magnetometer (VSM). Hysteresis loops at saturation field strength and a maximum field of 15.4 kA/m, as used in the case of SAR and remanence curve measurements, were determined. The switching field distribution S(H) was calculated from the remanence data using the following equation: $S\left(H\right)=\frac{dM_{r}\left(H\right)}{M_{rS}\times dH}$ with *M*_r_ being the field dependent initial remanence and *M*_rS_ as the saturation remanence [18].

## Results

**Figure 2:**
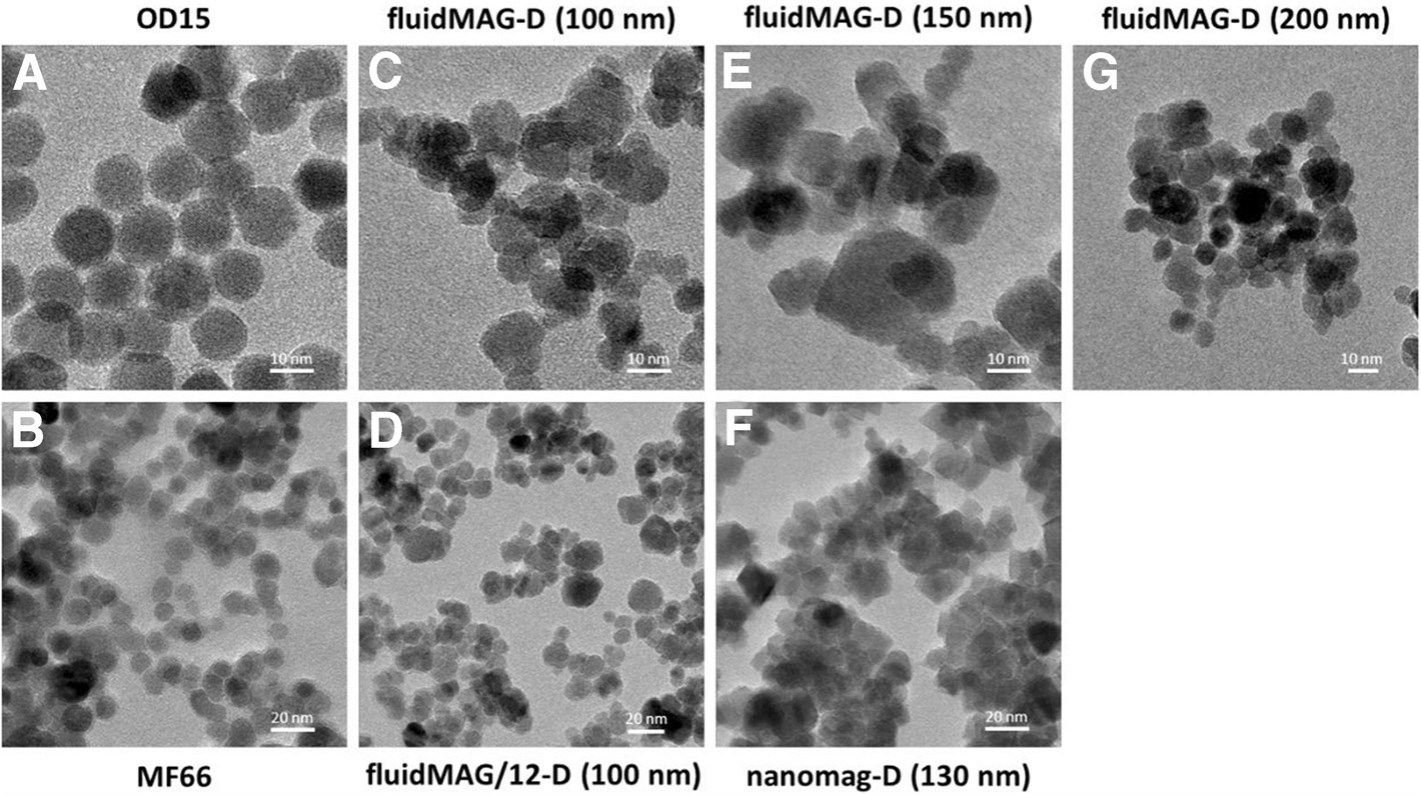
**TEM micrographs revealing different core characteristics of investigated magnetite MNP.** TEM micrographs of OD15 **(A)**, MF66 **(B)**, fluidMAG-D (100 nm) **(C)**, fluidMAG/12-D (100 nm) **(D)**, fluidMAG-D (150 nm) **(E)**, nanomag-D (130 nm) **(F)**, and fluidMAG-D (200 nm) **(G)** MNP. Magnification = ×75000 **(B, D, F)**. Magnification = ×160000 **(A, C, E, G)**.

HRTEM micrographs of single core OD15 and MF66 revealed a spherical structure of iron oxide cores with a narrow size distribution. The average core size was measured to be 15 ± 2 nm for OD15 and 12 ± 3 nm for MF66 MNP (Figure 2A, B). In contrast, fluidMAG-D, fluidMAG/12-D, and nanomag-D multicore MNP consisted of multiple irregularly clustered single cores different in size and shape (Figure 2C, D, E, F, G). Generally, agglomeration of single core MNP and the destruction of the spherical shape of multicore MNP clusters due to the sample preparation for HRTEM micrographs were observed.

DLS analysis of investigated MNP revealed polydispersity index (PDI) values which were smaller than 0.25, suggesting a relatively narrow particle size distribution. As expected, MNP *ζ*-potentials varied in dependence with the used MNP coating material (Table 1).

**Table 1:** Characteristics of investigated magnetite MNP

**Sample**	**Core structure**	**Coating**	**HRTEM**	**DLS**		***ζ*-potential**	**SAR (W/g Fe)**			**ILP (nHm**^**2**^**/kg)**		
			**Core size (nm)**	***Ø***_**hydr.**_**(nm)**	**PDI**	**(mV)**	**Fluid**	**Immobilized in 10% PVA**	**Immobilized in 1% agarose**	**Fluid**	**Immobilized in 10% PVA**	**Immobilized in 1% agarose**
fluidMAG-D (100 nm)	MC	Starch	65 to 75	110 ± 1	0.12	+3.1 ± 0.2	230 ± 13	98 ± 2	142 ± 2	2.2 ± 0.1	0.9 ± 0.0	1.4 ± 0.0
fluidMAG-D (150 nm)	MC	Starch	120 to 130	135 ± 1	0.15	+11.8 ± 0.4	362 ± 10	184 ± 18	285 ± 17	3.5 ± 0.1	1.8 ± 0.2	2.8 ± 0.2
fluidMAG-D (200 nm)	MC	Starch	165 to 175	170 ± 2	0.18	-3.1 ± 0.1	292 ± 54	143 ± 9	212 ± 7	2.8 ± 0.5	1.4 ± 0.1	2.1 ± 0.1
fluidMAG/12-D (100 nm)	MC	Starch	65 to 75	129 ± 4	0.25	0.8 ± 0.5	525 ± 36	332 ± 14	323 ± 14	5.1 ± 0.3	3.2 ± 0.1	3.1 ± 0.1
nanomag-D (130 nm)	MC	PEG300	115 to 125	163 ± 1	0.06	-31.9 ± 0.3	419 ± 20	170 ± 21	343 ± 20	4.1 ± 0.2	1.6 ± 0.2	3.3 ± 0.2
nanomag-D (130 nm)	MC	PEG300-COOH	115 to 125	156 ± 1	0.17	-16.9 ± 0.2	435 ± 15	241 ± 22	250 ± 11	4.2 ± 0.1	2.3 ± 0.2	2.4 ± 0.1
nanomag-D (130 nm)	MC	PEG300-NH2	115 to 125	165 ± 2	0.13	-20.4 ± 0.6	335 ± 24	169 ± 11	279 ± 8	3.2 ± 0.2	1.6 ± 0.1	2.7 ± 0.1
OD15	SC	DMSA	15	97 ± 0	0.21	-50.9 ± 2.0	658 ± 53	382 ± 39	550 ± 14	6.4 ± 0.5	3.7 ± 0.4	5.3 ± 0.1
OD15-PEG5000	SC	PEG5000	15	210 ± 1	0.21	-36.3 ± 0.6	417 ± 9	178 ± 16	291 ± 31	4.0 ± 0.1	1.7 ± 0.2	2.8 ± 0.3
OD15-PEG20000	SC	PEG20000	15	148 ± 1	0.23	-36.0 ± 0.8	413 ± 34	193 ± 31	387 ± 22	4.0 ± 0.3	1.9 ± 0.3	3.8 ± 0.2
MF66	SC	DMSA	12	108 ± 0	0.20	-52.4 ± 0.2	531 ± 23	224 ± 9	371 ± 38	5.2 ± 0.2	2.2 ± 0.1	3.0 ± 0.4
MF66-PAA	SC	PAA	12	93 ± 1	0.17	-42.3 ± 0.5	570 ± 5	262 ± 40	424 ± 38	5.5 ± 0.1	2.5 ± 0.4	4.1 ± 0.4
MF66-PEG10000	SC	PEG10000	12	92 ± 0	0.18	-40.4 ± 0.2	576 ± 13	248 ± 49	441 ± 32	5.6 ± 0.1	2.4 ± 0.5	4.3 ± 0.3
MF66-PEG10000-NH2	SC	PEG10000-NH2	12	204 ± 1	0.21	-37.1 ± 0.6	668 ± 3	314 ± 61	585 ± 32	6.5 ± 0.0	3.0 ± 0.6	5.7 ± 0.3

Depicted are the mean values with their corresponding standard deviations (*n* ≥3). *Ø*_hydr._, hydrodynamic diameter; MC, multicore; SC, single core; HRTEM, high-resolution transmission electron microscopy; DLS, dynamic light scattering; PDI, polydispersity index; SAR, specific absorption rate; PVA, polyvinyl alcohol; ILP, intrinsic loss power.

**Figure 3:**
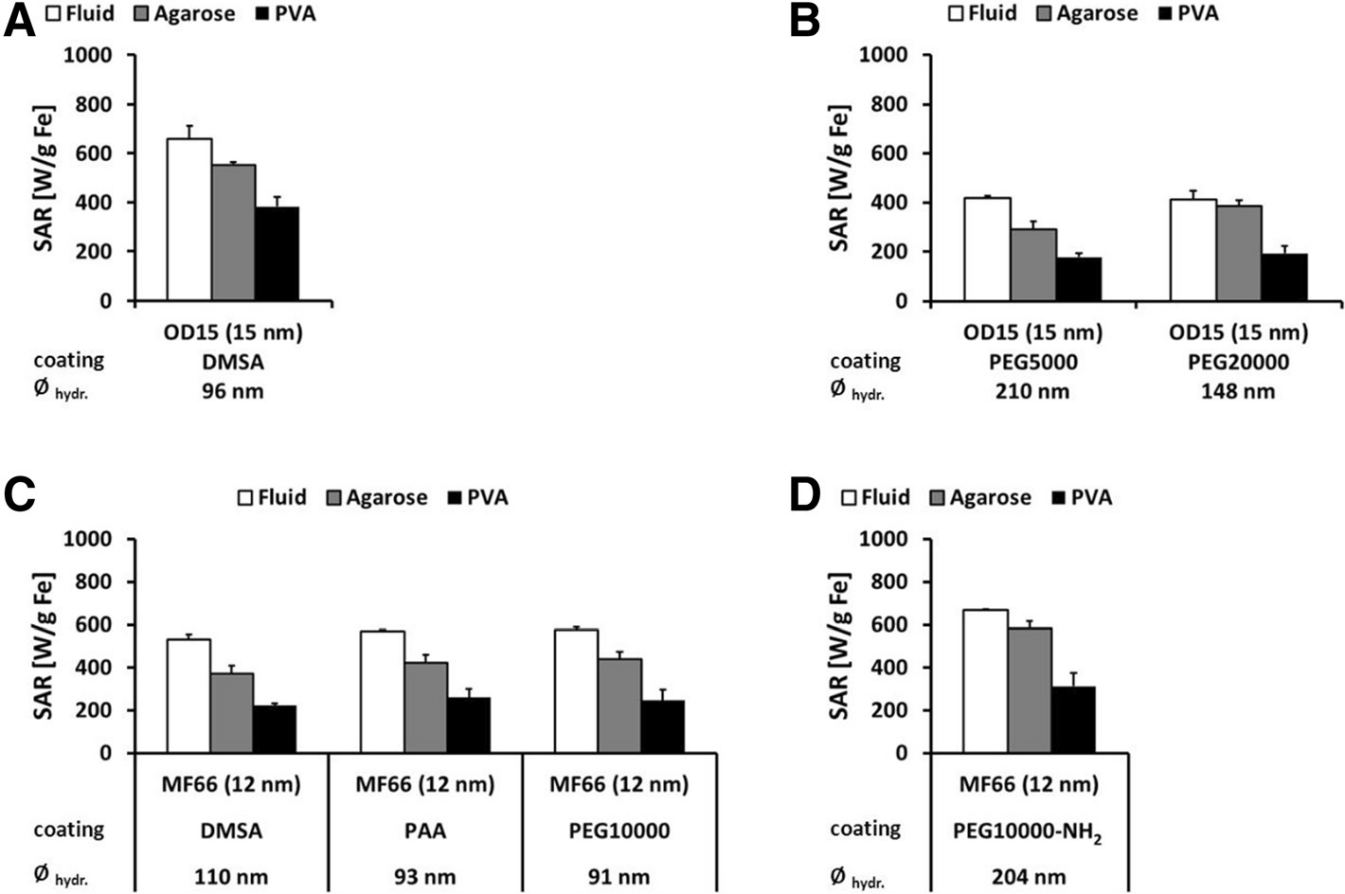
**Immobilization in PVA decreases the SAR of singlecore MNP OD15 and MF66.** Immobilization in PVA decreases the SAR of single-core MNP OD15 and MF66 by a factor of two compared to the respective water suspensions. SAR values of single core MNP in water suspension and immobilized in 1% agarose and 10% PVA. Additionally, hydrodynamic diameters (*Ø*_hydr._) for each MNP type are shown. Values in brackets indicate core size determined by TEM micrographs. Error bars indicate standard deviation of three independent measurements. OD15 coated with dimercaptosuccinic acid (DMSA) **(A)**; OD15 coated with polyethylenglycol (PEG) exhibiting different molecular weights **(B)**; MF66 coated with DMSA, polyacrylic acid (PAA), and PEG10000 **(C)**; and MF66 coated with PEG10000-NH_2_**(D)**.

In general, the immobilization of MNP in PVA resulted in decreased SAR values compared to MNP suspended in water, independently of their core structure, core size, and hydrodynamic diameter. Moreover, SAR values were reduced approximately by a factor of two after PVA immobilization in comparison to MNP suspended in water. The embedment of MNP in 1% agarose led to higher SAR values than the immobilization in 10% PVA, confirming a weaker immobilization of MNP in agarose than in PVA.The single core OD15 MNP, which were coated with DMSA and suspended in water, exhibited SAR values of 658 W/g Fe. Immobilization in 1% agarose decreased SAR to approximately 84% (550 W/g Fe). Lowest SAR values (382 W/g Fe) were observed after immobilization in PVA (Figure 3A). OD15 MNP with a comparable core size (15 nm) but coated either with DMSA or PEG showed almost the same ratio of SAR reduction after immobilization in PVA. Only marginal differences in SAR values of OD15 MNP with different hydrodynamic diameters and coated with PEG of different molecular weight (PEG5000: 210 nm, 5 kDa; PEG20000: 148 nm, 20 kDa) after PVA immobilization were observed. In contrast to a coating with PEG5000, the utilization of high molecular weight PEG (PEG20000) as surface coating led to comparatively higher SAR values after agarose immobilization (Figure 3B). The observed differences in absolute SAR values of fluidic OD15 MNP coated either with DMSA or PEG (Figure 3A, B) were related to the nature of the synthesis process of the uncoated MNP and varied among different batches. Hence, different SAR values after immobilization were seen. Nevertheless, the ratio of SAR reduction between different viscous immobilization media, especially in water and PVA, remained unchanged. Therefore, the coating with DMSA and PEG did not influence the SAR values of the fluidic MNP.

MF66 MNP with a single magnetic core of 12 nm and hydrodynamic diameters between 91 and 110 nm exhibited SAR values around 560 W/g Fe in water suspension; these values were not affected by the nature of MNP surface coating (DMSA, PAA, PEG10000). Immobilization of these MNP in agarose resulted in a reduction of SAR values to approximately 74% (approximately 412 W/g Fe), whereas PVA immobilization reduced the SAR further to 43% (approximately 244 W/g Fe) compared to MF66 MNP in water suspension (Figure 3C). Amino-functionalized MF66 MNP (MF66-PEG10000-NH_2_) with hydrodynamic diameters (204 nm) almost twice the hydrodynamic diameter of the other used MF66 MNP (approximately 98 nm) showed a reduction of SAR after immobilization in PVA to 47% (314 W/g Fe). In agarose, the SAR was reduced only to 87% (585 W/g Fe) in comparison to MNP in water suspension (668 W/g Fe; Figure 3D).

In comparison to the fluidic MF66 MNP coated with either DMSA, PAA, or PEG10000, higher SAR values of the fluidic MF66 MNP coated with PEG10000-NH_2_ were observed (Figure 3C, D). These differences were thought to be caused by minor variations in the synthesis process of the uncoated MNP (see OD15 in Figure 3A, B) rather than by the different coating materials.

**Figure 4:**
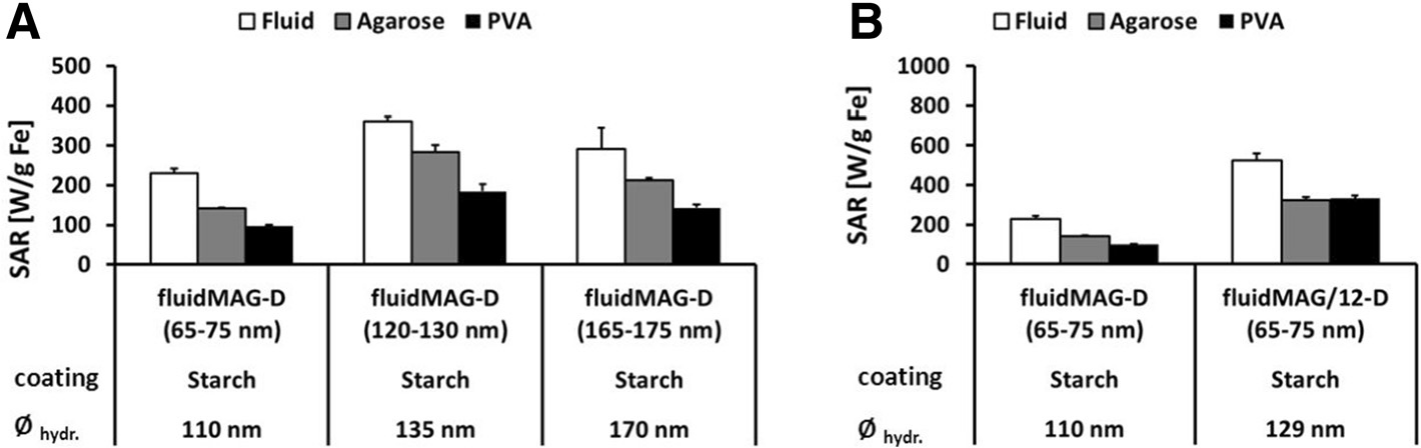
**Immobilization in polyvinyl alcohol decreases specific absorption rate (SAR) of multicore MNP fluidMAG-D by a factor of two.** SAR values of multicore MNP in water suspension and immobilized in 1% agar and 10% PVA. Additionally, hydrodynamic diameters (*Ø*_hydr._) for each MNP type are shown. Values in brackets indicate core size determined by TEM micrographs. Error bars indicate standard deviation of three independent measurements. fluidMAG-D coated with starch **(A)** and starch-coated 100-nm fluidMAG-D MNP with differently clustered cores **(B)**.

Immobilization of the multicore MNP (fluidMAG-D, core size: 65 to 175 nm, *Ø*_hydr._: 110 to 170 nm) in PVA led to a reduction of SAR values to approximately 48% of the fluidic samples (Figure 4A). Immobilization in agarose led only to a reduction to 71%. Interestingly, highest SAR values were observed for water suspended (362 W/g Fe) and immobilized MNP (agarose: 285 W/g Fe; PVA: 184 W/g Fe) with a hydrodynamic diameter of 135 nm (core size: 120 to 130 nm; Figure 4A).

The comparison of fluidMAG-D MNP and fluidMAG/12-D MNP (Figure 4B) comprising of the same core material/size, a comparable hydrodynamic diameter but a different clustering type, revealed a more than two times higher SAR of fluidMAG/12-D MNP (fluid: 525 W/g Fe; agarose: 323 W/g Fe; PVA: 331 W/g Fe) despite no major differences within the respective HRTEM micrographs (Figure 2C, D) being found. fluidMAG/12-D MNP showed almost no differences between the immobilization in agarose and PVA (Figure 4B). Both fluidic samples (fluidMAG-D and fluidMAG/12-D) exhibited a superparamagnetic behavior (*H*_C_ about 0.1 kA/m, remanence ration *M*_r_/*M*_S_ <0.008) as measured by VSM.

**Figure 5:**
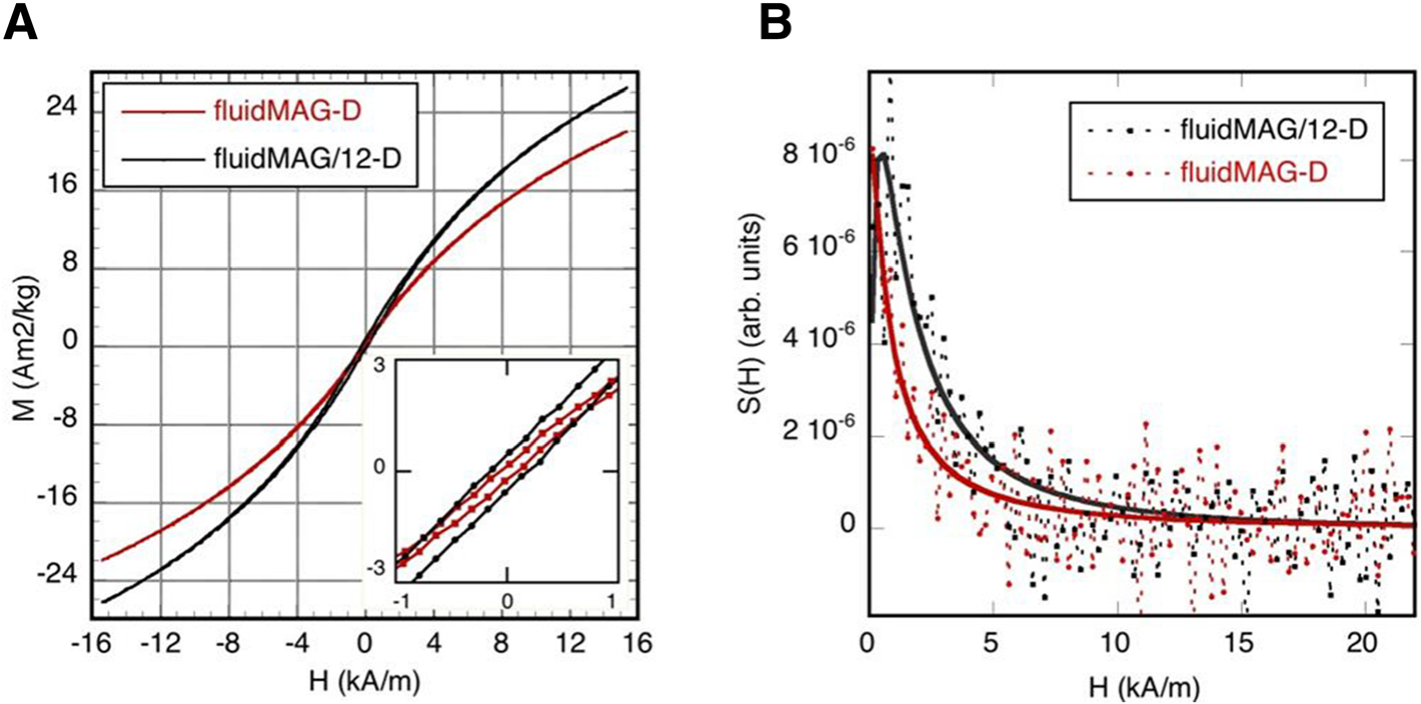
**Quasistatic magnetic measurements of immobilized MNP reveal differences in the magnetic behavior.** Minor magnetization loops of fluidMAG-D and fluidMAG/12-D MNP **(A)**. Insert: Magnification of the origin of ordinates reveals differences in hysteresis. Switching field distribution S(H) of fluidMAG-D and fluidMAG/12-D MNP **(B)**. Measurement points were fitted using log-normal-fit function.

**Figure 6:**
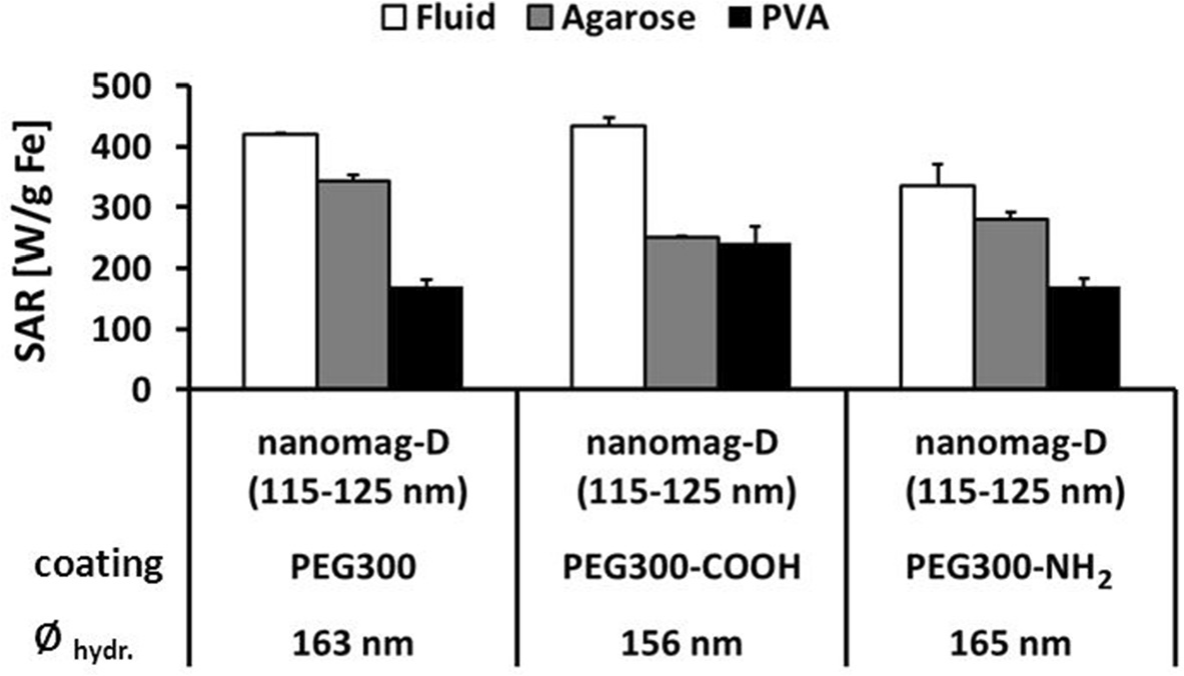
**Immobilization in polyvinyl alcohol decreases SAR of multicore nanomag-D MNP by a factor of two.** SAR values of multicore nanomag-D MNP with differently functionalized PEG300 in water suspension and immobilized in 1% agar and 10% PVA. Additionally, hydrodynamic diameters (*Ø*_hydr._) for each MNP type are shown. Values in brackets indicate core size determined by TEM micrographs. Error bars indicate standard deviation of three independent measurements.

Additionally, in order to characterize a possible non-superparamagnetic particle fraction, which might considerably influence the SAR, fluidic MNP were immobilized by their drying at 50°C and measured afterwards. For this kind of measurement, the use of agarose or PVA for MNP immobilization was not necessary. Hysteresis parameters of magnetization loops M(H) with *H*_max_ = 1,110 kA/m reveal small differences in the coercivity *H*_C_ and remanence ratio *M*_r_/*M*_S_ (fluidMAG/12-D: *H*_C_ = 0.32 kA/m, *M*_r_/*M*_S_ = 0.016; fluidMAG-D: *H*_C_ = 0.19 kA/m, *M*_r_/*M*_S_ = 0.009; Figure 5A). In the case of the minor loops with *H*_max_ = 15.4 kA/m, the corresponding data are as follows: fluidMAG/12-D: *H*_C_ = 0.17 kA/m, *M*_r_/*M*_S_ = 0.018; fluidMAG-D: *H*_C_ = 0.08 kA/m, *M*_r_/*M*_S_ = 0.008 (Figure 5A). A slight difference of the slope of the curves at zero field was obvious. Magnetization values were not considered since the amount of non-magnetic (diamagnetic) material in the dried samples (coating) is not known. Additionally, the switching field distribution S(H) was calculated. FluidMAG/12-D MNP showed, compared to fluidMAG-D MNP, a bigger fraction of MNP switching at ‘higher’ fields (*H* >0) but below the excitation field of 15.4 kA/m (Figure 5B).Analysis of nanomag-D MNP (core size: 5 to 15 nm) with hydrodynamic diameters between 156 to 165 nm and coated with differently functionalized PEG exhibited SAR values between 335 and 434 W/g Fe (Figure 6). The observed differences in absolute SAR values of fluidic nanomag-D MNP of the same size were related to the nature of the synthesis process of the uncoated MNP and varied among different batches.

As observed before, the highest reduction of the SAR values and, therefore, the highest immobilization was achieved by PVA usage. In the case of nanomag-D, SAR values were reduced in PVA to 41% (170 W/g Fe, PEG300), 50% (241 W/g Fe, PEG300-COOH), and 55% (169 W/g Fe, PEG300-NH_2_) compared to the MNP suspended in water. In contrast, the SAR after immobilization in agarose varied strongly and led only to a reduction of SAR to 82% (343 W/g Fe, PEG300), 83% (250 W/g Fe, PEG300-COOH), and 57% (279 W/g Fe, PEG300-NH_2_) compared to the fluidic MNP (Figure 6).

## Discussion

Our investigations showed a clear reduction of SAR values after immobilization in 1% agarose gels and 10% PVA hydrogels in comparison to fluidic samples for all investigated MNP types when using defined AMF conditions (*H* = 15.4 kA/m, *f* = 435 kHz). The field parameters used within this study are in accordance with earlier publications, reporting a suitability of frequencies up to 1,200 kHz and field amplitudes up to 31 kA/m for magnetic hyperthermia as well as magnetic thermoablation as cancer treatment [19-23]. Moreover, in previous *in vitro* and *in vivo* experiments, we could show that the used field conditions meet the requirements for magnetic heating without any ‘unspecific’ tissue heating due to eddy currents [2].

It is known that SAR values are highly dependent on the applied frequency and magnetic field strength. Therefore, a conversion of SAR into the parameter-independent ILP was performed, which is only valid within the linear response theory as long as the applied magnetic field is much smaller than the magnetic field leading to the saturation magnetization [6,24]. The AMF conditions employed during this study are expected to fulfill these requirements.

HRTEM micrographs revealed a partial agglomeration of MNP and the destruction of the spherical shape of MNP clusters, which were highly likely caused during the preparation of the samples (e.g., by drying processes of the MNP suspension) for HRTEM analysis, since a narrow size distribution of the fluidic MNP was validated by PCS measurements. These effects are, at least partly, caused by the shrinkage of the MNP coating. In general, with the HRTEM technique used within this work, none of the MNP coatings were visible due to a high amount of carbon in the particle coating, which leads to a low contrast to the TEM grid.

Within the performed study, the reduction of the SAR was more obvious after PVA than agarose immobilization, very probably due to the relatively large pore size of the agarose gels which results in an incomplete inhibition of Brownian relaxation. Righetti et al. and Ackers et al. reported of pore sizes of at least 141 nm for 1% agarose gels most likely allowing Brownian rotation of MNP especially with smaller hydrodynamic diameters [12,13]. Nevertheless, no influence of the hydrodynamic diameter on SAR values for agarose immobilization was found. A reason for this observation may be found in a disturbance of the formation of the final cross-linked gel structure by the presence of the MNP, resulting in pore sizes other than the values given above. Further on, it seems that only a small portion of the MNP were sufficiently immobilized, causing a partial inhibition of Brownian relaxation and therefore resulted in the observed minor reduction of SAR values after immobilization in 1% agarose gels.

Therefore, an overestimation of the SAR of MNP after measuring their SAR in fluidic suspension as well as in 1% agarose gels occurs, which is not realistic for cases of MNP immobilized in tumor tissues [7,25].

In contrast, the immobilization of MNP in PVA lead to a higher reduction of SAR values compared to agarose, most likely through stronger inhibition of Brownian relaxation, leaving Néel relaxation and hysteresis as the main contributor for MNP heating. The observed SAR reduction by an almost complete immobilization of the MNP mimics the *in vivo* situation where MNP are attached to cell membranes or are taken up into intracellular vesicles. In this context, Dutz et al. reported that up to 24 h after intratumoral MNP injection between 85% and 89% of administered MNP were immobilized to tumor tissue when comparing the coercivity and relative remanence of the injected MNP to MNP immobilized in gelatin [7]. As PVA was developed as a standard material in MRI for the determination of biomechanical characteristics of soft tissues, we assume its feasibility for simulating the immobilized state of MNP after *in vivo* application [17,26].

The comparison of single core and multicore MNP revealed higher absolute SAR values for single core MNP when using a field strength of 15.4 kA/m at a frequency of 435 kHz. This might be caused by a favorable hydrodynamic diameter of the used single core MNP leading to high Brownian relaxation losses in fluidic samples. When Brownian relaxation is hampered, immobilized multicore MNP own a higher coercivity than single core MNP and the applied field strength might be too low to exploit the SAR of such ferrimagnetic MNP within the investigated samples.

Although HRTEM micrographs revealed no obvious differences of the core structures of fluidMAG/12-D and fluidMAG-D MNP, they exhibited markedly different SAR values, which were thought to be related to differences in the clustering of the magnetic core of the MNP. In order to reveal the reason of the 2.3-fold higher SAR of fluidMAG/12-D MNP compared to fluidMAG-D MNP, magnetic characteristics were determined by VSM measurements. As one parameter, the switching field distribution S(H), should illustrate the possible influence of the biggest, i.e., hysteretic MNP in a sample with a certain size distribution, even if their fraction is small. Therefore, S(H) can be regarded as the distribution of the amount of MNP that switch their magnetization irreversibly at the field *H*. fluidMAG/12-D MNP revealed a bigger fraction of MNP switching at ‘higher’ fields (*H* >0) but below the excitation field of 15.4 kA/m than fluidMAG-D MNP, explaining the higher SAR value of the sample. However, the origin of this behavior is not known. In our case, a slightly changed size distribution of the initial iron oxide MNP (which is not detectable via HRTEM images) during the MNP synthesis or altered magnetic interaction by modified spatial MNP arrangements seems possible.

An underestimation of the SAR in the immobilized state could only be possible in a case of large single domain MNP showing ‘Stoner-Wohlfarth-like’ behavior at large field amplitudes [27]. However, this case can be excluded in the present work.

The surface charge of the MNP, referred to as *ζ*-potential, was observed to have no impact onto the SAR of (un-)functionalized single core or multicore MNP. Based on this finding, the immobilization seems to be rather of mechanical (adhesive binding) nature than an electrostatic one. Although we found no correlation between the *ζ*-potential and the SAR, the *ζ*-potential is crucial for cellular uptake [28-30].

Further on, the *ζ*-potential is thought to influence the degree of agglomeration and therefore the SAR of any given MNP. In this regard, a neutralization of the *ζ*-potential leads to a stronger tendency of agglomeration due to decreasing electrostatic repulsion forces between single MNP. This agglomeration affects the Brownian relaxation losses in liquid samples and thus also the resulting SAR. There will be an optimum of the size of MNP agglomerates leading to the highest SAR values for applied field parameters which is just an effect of MNP agglomeration and has no relation to the SAR of the single MNP within the fluid. But since for the real case of MNP applied to tissue Brownian relaxation is dramatically reduced, the change of magnetic properties due to exchange or dipole-dipole interactions is the dominant effect of agglomeration on SAR values. In short, agglomeration of superparamagnetic particles may lead to an increasing coercivity due to exchange interaction whereby the coercivity of agglomerates of ferrimagnetic MNP may decrease due to dipole-dipole interactions [1]. Since the coercivity of immobilized MNP is the main factor for resulting SAR, there will be a strong influence of agglomeration on SAR of immobilized MNP. To exclude potential artifacts of MNP agglomeration during experimental handling, MNP suspensions were treated with ultrasound before starting the measurement and immobilization steps. This strategy guaranteed the availability of proper MNP suspensions for several hours as confirmed by dynamic light scattering.

## Conclusions

Our investigation showed that the SAR of MNP in fluidic and immobilized state is only influenced by the specific characteristics of the MNP core including core structure and core size. Further on, the synthesis of MNP can influence the SAR of MNP exhibiting the same core size.

Immobilization in 10% PVA hydrogels led to a higher reduction of SAR compared to the usage of 1% agarose gels, indicating a higher degree of immobilization and therefore a stronger inhibition of Brownian relaxation. This most probably leaves Néel relaxation and hysteresis as the most contributing mechanism for heat generation. As assumed, we could successfully demonstrate that PVA is well suited to simulate the binding and immobilization state of MNP after *in vivo* application. As shown in this study, MNP immobilization in PVA can help to prevent an overestimation of SAR values measured under ferrofluidic conditions, allowing a more realistic estimation of the SAR in the *in vivo* situation.

Moreover, no influence of coating material on SAR was found for fluidic and immobilized MNP. Especially, no correlation between the functionalization and/or the surface charge (*ζ*-potential), as well as the hydrodynamic diameter, was found for fluidic or immobilized MNP. However, the coating material and the resulting surface charges are of high importance for blood half-life, cellular uptake, and biocompatibility.

## Competing interests

The authors declare that they have no competing interests.

## Authors’ contributions

The experimental design and particle measurements (except for VSM measurements) were carried out by RL and MS. RL and MS were the main authors and contributed equally to the draft of the manuscript. RM performed the VSM measurements and helped to draft the manuscript. SD participated in the draft of the manuscript. IH supervised the design and helped to draft and supervise the manuscript. UT supervised the manuscript. All authors read and approved the final manuscript.

## Authors’ information

RL and MS are presently PhD students at the Division of Diagnostic and Interventional Radiology at the University Hospital of the Friedrich-Schiller-University Jena. SD (PhD) is Head of the Magnetic Nanoparticles Group at the Institute of Biomedical Engineering and Informatics of the University of Technology Ilmenau. RM (PhD) is a Research Scientist at the Department of Nanobiophotonics of the Leibniz Institute of Photonic Technology Jena. UT (MD) is Professor and Chair at the Division of Diagnostic and Interventional Radiology at the University Hospital of the Friedrich-Schiller-University Jena. IH (PhD) is Professor and Head of the Department of Experimental Radiology at the Division of Diagnostic and Interventional Radiology at the University Hospital of the Friedrich-Schiller-University Jena.
